# Prognostic significance of the sequential detection of circulating melanoma cells by RT–PCR in high-risk melanoma patients receiving adjuvant interferon

**DOI:** 10.1038/sj.bjc.6600419

**Published:** 2002-07-02

**Authors:** H Gogas, G Kefala, D Bafaloukos, K Frangia, A Polyzos, D Pectasides, D Tsoutsos, P Panagiotou, J Ioannovich, D Loukopoulos

**Affiliations:** Research Laboratory of 1st Department of Medicine, University of Athens; Aghiou Thoma 17, Goudi, Athens 11 527, Greece; Hellenic Cooperative Oncology Group (HeCOG), Data office, Laskaridou 1, Athens 11 524, Greece

**Keywords:** tyrosinase mRNA, interferon, melanoma, RT–PCR

## Abstract

The purpose of this study was to address the prognostic significance of circulating melanoma cells by reverse transcriptase-polymerase chain reaction in the peripheral blood of stage IIB and III melanoma patients on high-dose adjuvant interferon at multiple sequential time points from initiation of treatment. Tyrosinase mRNA in peripheral blood from these patients was assayed by reverse transcriptase polymerase chain reaction prior to initiation of adjuvant interferon, at completion of 1 month of intravenous interferon and at 3 monthly intervals until progression. Four hundred and eighteen blood samples from 60 melanoma patients were analysed. The median follow-up time calculated from the time of inclusion in the study was 23 months (range 2–38 months). Tyrosinase mRNA in blood was detected in 42 (70%) of 60 patients: 16 (76%) of 21 stage IIB patients and 26 (66%) of 39 stage III patients. The presence of tyrosinase mRNA in blood was correlated with a shorter disease-free survival (*P* : 0.03) and in multivariante analysis was an indepent prognostic factor for relapse. Patients who seroconverted to a negative reverse-transcriptase-polymerase chain reaction after induction treatment had a significantly lower probability of recurrence. The presence of circulating melanoma cells is a marker of a high relapse risk and shorter disease-free survival whether detected postoperatively or during follow-up. Tyrosinase mRNA amplification by reverse-transcriptase-polymerase chain reaction may be a useful tool for monitoring the efficacy of adjuvant treatment in stage IIB and III melanoma patients.

*British Journal of Cancer* (2002) **87**, 181–186. doi:10.1038/sj.bjc.6600419 www.bjcancer.com

© 2002 Cancer Research UK

## 

For detecting tumour cells in melanoma patients reverse transcription-polymerase chain reaction (RT–PCR) has been used to identify tyrosinase, a key enzyme in melanin biosynthesis, in the circulation of patients with disseminated disease (Smith *et al*, 1991). The tyrosinase gene is actively expressed only in melanocytes and melanoma cells (Kwon *et al*, 1987) and as melanocytes cannot be detected in the circulation, the detection of tyrosinase RNA indicates the presence of melanoma cells.

The relationship between circulating tumour cells and the development of secondary disease is not fully understood. The presence of such cells in peripheral blood samples from early stages melanoma could indicate a dissemination of the tumour cells, and thus a high risk of metastasis. If confirmed circulating melanoma cells could represent a new prognostic marker with which to predict clinical behaviour, assess tumour progression, and improve patient management.

However, different research groups reported conflicting results on the sensitivity and clinical value of tyrosinase RT–PCR (Foss *et al*, 1995; Stevens *et al*, 1996; Jung *et al*, 1997; Glaser *et al*, 1997). In patients with localised disease (stages I and II) the percentage of blood samples that test positive for tyrosinase varies greatly ranging from 0 (Kunter *et al*, 1996) to 45% (Reinhold *et al*, 1997). Blood samples from untreated patients with advanced disease show remarkable differences in tyrosinase-positive rates, which range from 0 to 44% (Battayani *et al*, 1995) for stage III patients and from 13% (Pittman *et al*, 1996) to 100% for stage IV patients (Brossart *et al*, 1993). Some groups have found that RT–PCR for tyrosinase maybe a prognostic marker in both early and late stages of disease. In one study PCR detection of circulating melanocytes was considered as a marker for rapid postoperative relapse after node dissection in patients with melanoma and regional node metastases, for short-term relapse in high-risk disease-free patients and for rapid and severe progression in patients with distant metastases (Battayani *et al*, 1995).

Although most of the studies have been focused on the detection of circulating melanoma cells at the time of initial diagnosis they can also be detected in blood months or years after primary tumour diagnosis (Curry *et al*, 1998).

However the biological and clinical implications that may be associated with the presence of circulating malignant cells during the follow-up of melanoma patients are not clear. To address the prognostic implications of the presence of late circulating melanoma cells, we decided to investigate the presence of tyrosinase mRNA by RT–PCR in the peripheral blood of stage IIB and III melanoma patients on high-dose adjuvant interferon a-2β at multiple sequential time points from initiation of treatment.

## MATERIALS AND METHODS

### Patients

Patients with a histologically documented diagnosis of cutaneous melanoma, stage IIB or III according to the American Joint Committee on Cancer (AJCC) guidelines were included in the study. Stage was defined on a pathological basis. Patients underwent sentinel node dissection. Any patient with a positive SLN was required to undergo a complete lymphadenectomy. All patients were initiated on high-dose inteferon according to Hellenic Cooperative Oncology Group protocol (Gogas *et al*, 2000) 2 months after initial surgery, or 1.5 months after therapeutic lymph node dissection for regional lymph node recurrence. Oral informed consent was obtained from each patient. Blood for tyrosinase RT–PCR was drawn at the same time that blood extraction was performed for previous established follow-up: the first 20 ml of blood collected were used for standard biochemistry and cell blood counts, and the following 15 ml were used for RT–PCR studies. Blood samples were obtained prior to induction treatment, at completion of 1 month intravenous interferon, and at 3 monthly intervals until progression. The clinical outcome of patients was prospectively followed. Clinical staging consisted of medical history, physical exam, cell blood count, blood biochemistry at 3 monthly intervals, chest X-ray and liver ultrasound at 6 monthly intervals. Other complimentary exams were performed if they were clinically indicated. Patients with a positive RT–PCR were not subjected to a more intensive follow-up than were those with a negative RT–PCR and no clinical decisions were made based on the results of the RT–PCR assay. Negative controls were healthy subjects and patients with other malignancies.

### Samples - RNA extraction

Twelve to 15 ml of blood were collected in EDTA anticoagulant from each patient. The mononuclear cell fraction of peripheral blood was isolated by ficoll gradient centrifugation (Boyum, 1968). Total RNA was isolated from the mononuclear cell fraction by guanidium thiocyanate extraction (Chomczynski and Sacchi, 1987). The extracted RNA was dissolved in ddH20. Integrity was evaluated by assesing the 18 S and 28 S ribosomal RNA bands in 2% ethidium-bromide-stained agarose gel. The amount of RNA was calculated by spectrophotometric analysis.

### Complementary DNA synthesis

For the complementary DNA (cDNA) synthesis 1 μg of RNA was incubated for 5 min at 65°C with 3 μg Random Primer oligonucleotides (Gibco-BRL), in a total volume of 13 μl. One mM of each deoxynucleotide triphospate (dNTP) (New England Biolabs), 1× First Strand Buffer and 200 U Moloney Murine Leukemia Virus Reverse Transcriptase (M-MLV RT) (Gibco BRL), were added to a final volume of 20 μl. cDNA synthesis was performed for 1 h at 37°C, followed by enzyme denaturation at 95°C for 2 min.

The Retinoic Acid Receptor a (RARa) gene was used as an internal control for assessing the quality of the cDNA. Effective amplification using the primers RaR6 and RaR8 confirmed the quality of the samples.

### PCR method

Ten μl of the previously obtained cDNA sample were amplified in the first PCR round using 100 pmol of each primer HTYR1 (sense: TTG, GCA, GAT, TGT, CTG, TAG, CC) and HTYR2 (antisense: AGG, CAT, TGT, GCA, TGC, TGC, TT) (Smith *et al*, 1991).

The reaction mixture also contained 0.2 mM of each dNTP, 1×PCR buffer, 2 mM MgCl_2_ and 2 U Taq DNA Polymerase (Gibco BRL) in a final volume of 50 μl.

The PCR began with one cycle of 5 min at 94°C for template denaturation, followed by 30 cycles of 1 min denaturation at 94°C, 1 min at 55°C for primer annealing and 1 min for polymerase extension at 72°C. The reaction was terminated with a 10 min extension at 72°C. Five μl of a 1 : 100 dilution of the first-round PKR product were reamplified using the nested primers HTYR3 (sense: GTC, TTT, ATG, CAA, TGG, AAC, GC) and HTYR4 (antisense GCT, ATC, CCA, GTA, AGT, GGA, CT) (1) in a 25 μl final volume, for a further round of 30 cycles under the same conditions as the first PCR round.

The outer primers amplify a PCR, product of 284 base pairs, while the nested primers amplify a fragment of 207 base pairs. The PCR products were visualised in ethidium bromide-stained 3% agarose gel.

To determine the sensitivity of the assay serial dilutions of 10^6^ to 1 SK-Mel 28 cells in 10^7^ normal mononuclated peripheral blood cells, were prepared. RT–PCR was performed using 1 μg of total RNA from each dilution. The sensitivity of the method was high enough to detect 10 SK-Mel 28 cells in 10^7^ normal cells. Southern blot was performed to confirm that our RT–PCR amplified products were indeed from tyrosinase.

### Statistical analysis

Relapse free survival was calculated from the initiation of treatment to the date relapse of the disease (RFS) was firstly documented and overall survival (OS) from initiation of treatment to the date of death or last contact. All patients without the corresponding event by 30/9/00 were considered censored. Sensitivity was calculated as the proportion of relapsed patients with positive RT–PCR. Specificity as the proportion of non-relapsed patients with negative RT–PCR. Positive predicted value as the proportion of positive RT–PCR patients with relapse. Negative predicted value as the proportion of negative RT–PCR patients without being relapsed.

The χ^2^ test were used to test the association between RT–PCR status and stage, Breslow, Clark, ulceration, regression, lymph node status, primary tumour location, sex, age. The Kaplan–Meier method was used to calculate RFS and OS curves, while the log-rank test was used to compare time to event distributions. Multivariate analysis were performed with Cox proportional hazard models in order to indicate independent prognostic factors for survival and relapse-free survival. A backward selection procedure identified the subclass of significant variables among the following: stage (IIB *vs* III), Breslow (0–2 *vs* 2.1–4>4), Clark (II–III *vs* IV *vs* V), ulceration (No *vs* Yes), regression (No *vs* Yes), lymph node status (No *vs* Yes) primary tumour location, sex (male *vs* female), age (<52 *vs* 52). The significant factors were kept in the model if the maximum likelihood ration criterion had a *P*-value below 0.10. All reported *P*-values are two-sided.

Calculations were made using each patient. A negative patient is a patient whom no positive result has ever been obtained and a positive patient, is a patient in whom at least one positive test has been obtained. As all patients with positive sentinel lymphnode had undergone lymphadenectomy, no nodal relapses were seen in our study population and relapses are distant metastasis.

## RESULTS

### Patients

Four hundred and eighteen blood samples from 60 melanoma patients were tested for tyrosinase mRNA in blood by RT–PCR. The median number of RT–PCR tests per patient was seven (range 2 to 13). Blood from eight controls (four patients with breast cancer and four patients with colon cancer) was also examined. Patient's characteristics are described in Table 1

**Table 1:**
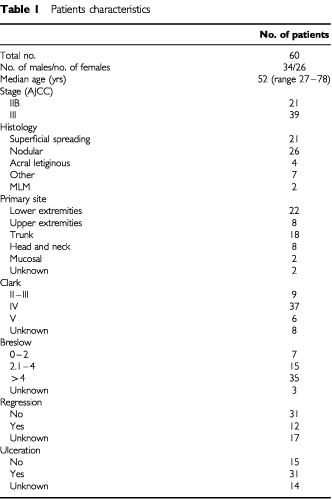
Patients characteristics

. The patients were followed for a median time (calculated from the inclusion in the study) of 23 months (range 2, −38+months, 95% CI 17.4–28.6).

### RT–PCR status and relapses

**Figure 1:**
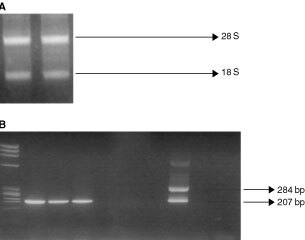
(**A**) 28 S and 18 S rRNA bands. Total RNA extraction as visualised in ethidium bromide stained 2% agarose gel. (**B**) Ethidium bromide-stained gel visualisation of RT–PCR products showing a 207 bp band in the positive samples. Lane 1. Molecular Weight Marker (ΦX174 RF DNA *Hae*III Digest). Lanes 2–4. Positive samples (three melanoma patients). Lanes 5–7. Negative samples (three melanoma patients). Lane 8. Positive control (SK-mel 28 cell line). Lane 9. Negative control (healthy donor). Lane 10. Negative control (sample without cDNA).

RNA samples that showed the 28 S and 18 S ribosomal band were considered eligible for the study (Figure 1A

). Samples showing a band of 207 bp after a second round of amplification with nested primers are considered positive (Figure 1B). Using the same RT–PCR technique no false-positive results among 16 samples from non melanoma control patients (eight healthy subjects and eight patients with other cancers). Hence, no illegitimate transcription in the haematopoietic cells was observed among the non melanoma controls with the experimental conditions used.

Here 105 (25%) of 418 blood samples were positive for tyrosinase. Circulating melanoma cells were detected in 42 of 60 patients. There was no correlation between RT–PCR status and age, sex, Breslow, Clark, ulceration, regression, lymph node involvement and stage. Prior to initiation of induction treatment with intravenous interferon 30 patients had negative RT–PCR, and 30 had positive. After completion of 1 month of induction treatment 16 patients were tested with a negative RT–PCR and 14 continued to test positive. In the group of patients who initially had a negative RT–PCR result, 21 had a second negative result and nine seroconverted, to a positive result, after 4 weeks of intravenous high-dose interferon. Eighteen patients had sequential negative RT–PCRs and 42 patients had one or more positive blood samples for tyrosinase. With a median follow-up of 23 months 29 patients have recurred and 13 have died from melanoma dissemination. One patient has died, remaining disease-free. Eighteen patients had a positive RT–PCR at initiation of adjuvant treatment, eight tested positive during follow-up and finally three had sequential negative RT–PCRs even at relapse. The sites of relapse for these RT–RCR negative patients were subcutaneous tissue (1), lymph nodes (2). The median interval between the detection of a positive RT–PCR result and the diagnosis of clinical relapse was 21 months (range 1–36 months, 95% CI 11.7–20.3). In the group of patients with no evidence of disease, 15 had multiple negative RT–PCRs, nine had a positive sample prior to induction therapy and negative results during treatment and further follow-up, two had initially two positive RT–PCRs (one prior to induction therapy and at completion of intravenous interferon) and negative samples during maintenance treatment and further follow-up, and finally five patients had more than one positive sample during follow-up (Table 2

**Table 2:**
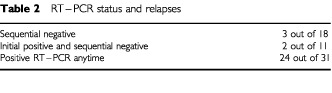
RT–PCR status and relapses

).

**Figure 2:**
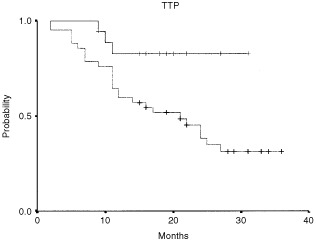
RT–PCR results and relapse-free survival in stage IIB and III melanoma patient calculated by the Kaplan–Meier method.

**Figure 3:**
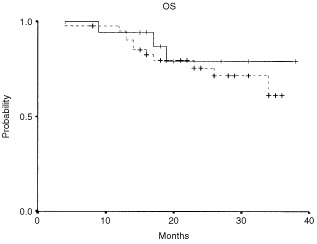
RT–PCR results and overall survival in stage IIB and III melanoma patients calculated by the Kaplan–Meier method.

The RT–PCR positivity by tyrosinase mRNA in blood during follow-up of patients with melanoma significantly correlated with shorter disease-free survival (*P*: 0.029). The median disease-free survival was 21 months (95% CI 12.7–29.3) for the RT–PCR positive group. The median disease-free survival was not reached for the group of patients testing negative for tyrosinase in blood (Figure 2

). Median overall-survival was not reached for either group of patients (Figure 3

).

In univariante analysis for relapse free survival only RT–PCR status was correlated with relapse (*P*: 0.03) (Table 3

**Table 3:**
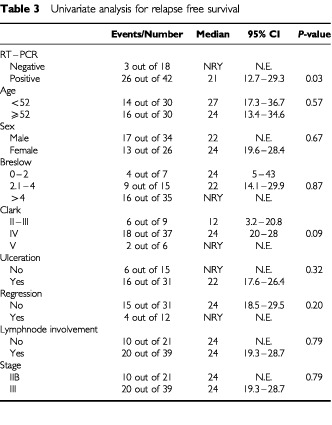
Univariate analysis for relapse free survival

). In univariante analysis for survival no variable was found to be correlated with RT–PCR status (Table 4

**Table 4:**
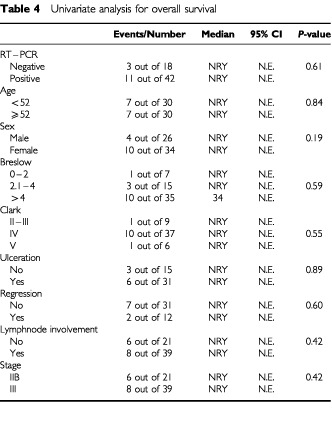
Univariate analysis for overall survival

). In multivariante analysis (Cox regression analysis) RT–PCR status was the only independent prognostic factor for relapse (β=1.3, 95% CI=0.1–2.5, *P*-value=0.03). No independent prognostic factor was found for overall survival. Multivariate analysis was performed without the factors ulceration and regression because of large numbers of unknown values and the results were exactly the same as these mentioned above.

The sensitivity (probability that a patient who tests positive has recurrent disease) was 89.66% (95% CI=72–98%) and the specificity (probability that a patient who tests negative remained disease-free) was 48.39% (95% CI=30–67%). The positive predictive value was 61.90% (95% CI=46–76%) and the negative predictive value was 83.33% (95% CI=59–96%).

There was a statistically significant difference in the proportion of patients remaining disease-free and patients with negative (15 out of 18=83%) and those with positive RT–PCR (16 out of 42=38%) (*P*=0.002). There was evidence that the proportion (9 out of 11=82%) of seroconverted patients (patients who had a positive RT–PCR prior to induction therapy and negative RT–PCRs during treatment and further follow-up) remaining disease-free was statistically significantly higher than that (7 out of 31=23%) of patients being positive (*P*=0.001). Furthermore, there was not any evidence that the proportion of seroconverted patients remaining disease-free was statistically significantly higher than that (15 out of 18=83%) of patients being negative (*P*=0.92).

## DISCUSSION

Conflicting results have been obtained by various research groups using tyrosinase reverse transcription–polymerase chain reaction (RT–PCR) for detecting micrometastases in the blood of melanoma patients, with positive results ranging from 0 to 100% in disseminated melanoma. Methological differences in the processing of blood samples may in part account for these discrepancies. The prognostic significance of the detection of circulating melanoma cells by RT–PCR has been evaluated in several studies. In high-risk but apparently disease-free patients, the risk of relapse within the next 6 months was 3.8 times higher after a positive test result, than after negative PCR test (*P*: 0.002), and in patients with distant metastases after a positive PCR, rapid progression was four times more likely than slow progression or stable disease (Battayani *et al*, 1995). In another study patients with a positive PCR showed a 2.4-fold increased risk for relapse compared with PCR-negative patients (Schittek *et al*, 1999). The Melanoma Cooperative Group performed RT–PCR using tyrosinase, p 97, MUC 18, and MelanA/MART1 as gene markers in 235 patients with either localised or metastatic melanoma. Univariante analysis showed a significant correlation between risk of recurrence (evaluated in stage I, II and III patients and increasing number of PCR-positive markers (*P*: 0.0002). Logistic regression multivariante analysis indicated that each single marker (except tyrosinase) and more especially, the presence of PCR-positive markers remained a statistically independent prognostic factor for tumour progression (Pialmieri *et al*, 1999). Finally in one study conducted in long-term clinically disease-free melanoma patients, regardless of their initial clinical stage, the presence of late circulating melanoma cells was significantly associated with a subsequent high risk of relapse and death (Mellado *et al*, 1999).

Here we specifically studied high-risk melanoma patients to assess the clinical significance of the sequential detection of circulating melanoma cells. The first finding from our data was that one or more positive tests for tyrosinase mRNA in blood during long-term follow-up of patients with melanoma was significantly correlated with a higher risk of relapse, shown both in univariante and multivariante analysis but not a higher risk of death from melanoma dissemination as found in other studies (Mellado *et al*, 1999). Maybe longer follow-up is warranted as median overall survival has not been reached by either tyrosinase positive or negative group. In another study (Curry *et al*, 1998) where blood samples from 276 melanoma patients were tested for tyrosinase and MART-1 at multiple sequential time points for at least 2 years after surgery, a low incidence of RT–PCR positivity was observed even up to 2 years after surgery. This occurred both in patients who had been continuously positive and in patients who had developed positive results after several negative results. Given the short follow-up of the patients, the authors could not assess, the prognostic significance of their late RT–PCR positive results and consequently could not exclude that these were false positive results. However the poor prognosis observed in our group of patients with a positive RT–PCR presented here suggest that these positivities are due to the presence of melanoma cells in the circulation in disease-free patients rather than those false-positive tests. This is also supported by the worst prognosis reported in other studies evaluating the role of the late circulating melanoma cells in long-term disease-free patients (Mellado *et al*, 1999).

Only three out of 18 patients with sequential negative RT–PCRs recurred (16.6%), a difference which was statistically significant. Two out of 11 patients who tested positive prior to induction therapy and subsequently had negative RT–PCRs during treatment and further follow-up, recurred which was again statistically different.

On the basis of this observation, it is reasonable to speculate that the late circulating melanoma cells detected in our patients can be shed from secondary organs. If this were true then the presence of circulating malignant cells in the setting of high-risk melanoma patients would be a marker of the existence of micrometastatic disease.

Patients who seroconverted to a negative RT–PCR result after induction treatment with intravenous interferon, had a significantly lower probability of recurrence. Even though, this is not a randomised study, and there were no untreated patients, and one could speculate that the conversion was a consequence of greater time after surgery, the surgery in this case either dislodging tumour cells or removing the source of tumour cells, the lower probability of recurrence strongly argues that the reversion was a consequence of interferon therapy. These results cannot be extrapolated to the general stage II and III population as IFN may have interfered with the results.

A negative RT–PCR result did not exclude tumour progression, as patients with sequential negative RT–PCRs developed metastases. Reasons proposed by other investigators might be that (i) melanoma cells are shed only intermittently from the tumours into the circulation; (ii) the lymph nodes work as a filter for tumour cells in the circulation, and (iii) the sensitivity of the RT–PCR is still suboptimal and not all melanoma cell transcripts can be detected (Schittek *et al*, 1999). Another probable explanation for the three patients who developed recurrent disease despite negative RT–PCR tests is that the recurrent melanoma was tyrosinase negative. Unfortunately tumour specimens from recurrences were not tested for tyrosinase expression.

In conclusion our data suggests that circulating melanoma cells are markers of a high relapse risk and shorter disease-free survival whether detected postoperatively or during follow-up. Tyrosinase mRNA amplification by RT–PCR may be a useful tool for monitoring the efficacy of adjuvant treatment in stage IIB and III melanoma patients.
